# CCL5–Glutamate Cross-Talk in Astrocyte-Neuron Communication in Multiple Sclerosis

**DOI:** 10.3389/fimmu.2017.01079

**Published:** 2017-09-04

**Authors:** Anna Pittaluga

**Affiliations:** ^1^Department of Pharmacy, DIFAR, Pharmacology and Toxicology Section, University of Genoa, Genoa, Italy; ^2^Center of Excellence for Biomedical Research, University of Genoa, Genoa, Italy

**Keywords:** CCL5, glutamate, nerve endings, multiple sclerosis, experimental autoimmune encephalomyelitis mice, release

## Abstract

The immune system (IS) and the central nervous system (CNS) are functionally coupled, and a large number of endogenous molecules (i.e., the chemokines for the IS and the classic neurotransmitters for the CNS) are shared in common between the two systems. These interactions are key elements for the elucidation of the pathogenesis of central inflammatory diseases. In recent years, evidence has been provided supporting the role of chemokines as modulators of central neurotransmission. It is the case of the chemokines CCL2 and CXCL12 that control pre- and/or post-synaptically the chemical transmission. This article aims to review the functional cross-talk linking another endogenous pro-inflammatory factor released by glial cells, i.e., the chemokine Regulated upon Activation Normal T-cell Expressed and Secreted (CCL5) and the principal neurotransmitter in CNS (i.e., glutamate) in physiological and pathological conditions. In particular, the review discusses preclinical data concerning the role of CCL5 as a modulator of central glutamatergic transmission in healthy and demyelinating disorders. The CCL5-mediated control of glutamate release at chemical synapses could be relevant either to the onset of psychiatric symptoms that often accompany the development of multiple sclerosis (MS), but also it might indirectly give a rationale for the progression of inflammation and demyelination. The impact of disease-modifying therapies for the cure of MS on the endogenous availability of CCL5 in CNS will be also summarized. We apologize in advance for omission in our coverage of the existing literature.

## Introduction

The immune system and the central nervous system (CNS) cross-talk, and this interaction are pivotal to the onset and the progression of central neurodegenerative diseases (i.e., Alzheimer’s disease and amyotrophic lateral sclerosis), as well as in classic autoimmune-inflammatory disorders [i.e., multiple sclerosis (MS)]. Although inflammation probably does not represent an initiating factor, new evidence suggests that pro-inflammatory molecules contribute to the derangement of chemical synapses favoring disease progression (1). In fact, a number of endogenous molecules (i.e., cytokines and chemokines) released by the immunocompetent cells as well as by activated astrocytes control chemical transmission at active synapses, affecting the main functions of these cells including transmitter release and second messenger production. These endogenous molecules are main transducers of the pathological “glial to neuron” cross-talk (1–5).

### Chemokines: General Considerations

In mammals, the chemokine (chemotactic cytokines) universe comprised of approximately 50 endogenous small (8–14 kDa) peptides released by immune cells and 20 receptors (4, 5). Chemokines have a tertiary structure highly conserved and are subdivided into four groups (namely the CC, the CXC, the CX_3_C, and the C subfamilies) based on the relative positions of two conserved cysteine residues near the N-terminus (6). Chemokines act at chemokine receptors that are seven transmembrane domain *Pertussis* toxin (PTx)-sensitive, G-protein-coupled receptors (GPCRs), which depending on the G protein involved, trigger enzymatic cascade of events controlling several intraterminal pathways, mostly controlling Ca^2+^ ions mobilization, intraterminal phosphorylative pathways, and small Rho GTPases signaling (7). First identified for their ability to mediate leukocyte chemo-attraction in inflammatory and autoimmune diseases (8), chemokines and their receptors are now recognized as a promiscuous and redundant system of signaling interactions and mutual binding relevant to inflammation, immunity and neuropathology. Most of the chemokine receptors bind more than one ligand, and several chemokines activate more than one receptor, accounting for the numerical mismatch among chemokines and relative receptors. In particular, CCR1, CCR3, and CCR5 are promiscuous receptors for different chemokines including CCL3 (macrophage inflammatory protein 1-alpha), CCL5 [Regulated upon Activation Normal T-cell Expressed and Secreted (RANTES)], and CCL7 (monocyte chemotactic protein-3). All these aspects have been largely documented in previous articles (3, 4, 9–19) and will not be further detailed.

### CCL5

CCL5 plays a main role in inflammatory diseases and in cancer, because of its ability to control the movements of memory T lymphocytes, monocytes macrophages, and eosinophils (4–7). Evidence has been provided also showing a role of this chemokine in CNS diseases secondary to viral infections, such as the acquired immuno deficiency syndrome-related dementia, or involving neuro-inflammatory processes, such as MS, Alzheimer dementia, and Parkinson’s disease (6, 12, 13, 20–23).

**Figure 1:**
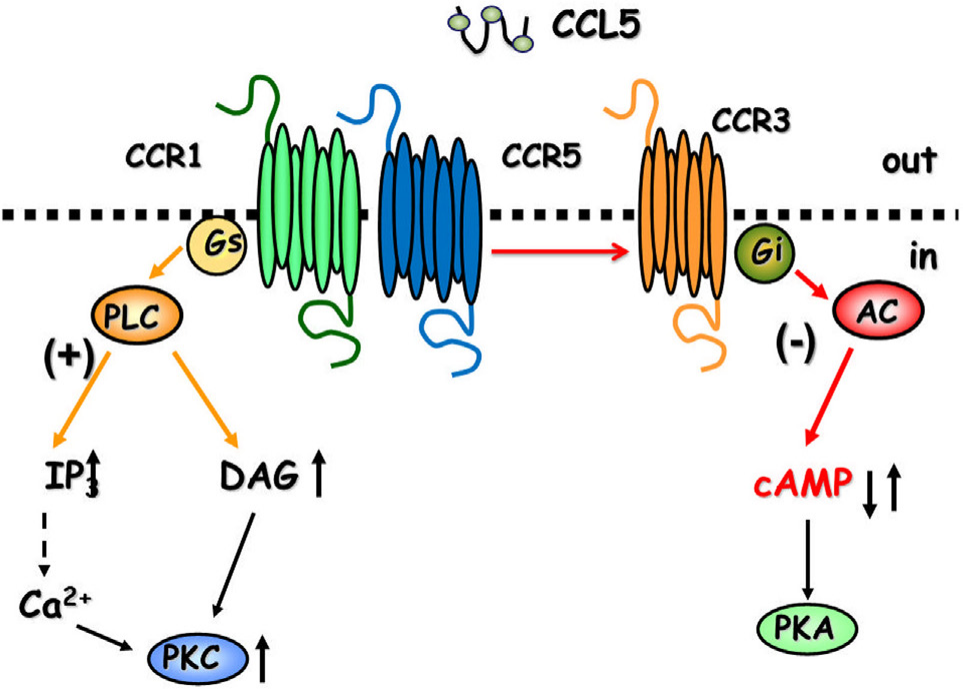
The cartoon summarizes the intraterminal pathways involved in the facilitation and in the inhibition of glutamate exocytosis in mammals glutamatergic nerve endings. CCR1/CCR5 heterodimers couple to G proteins leading to phospholipase C (PLC) translocation, hydrolysis of membrane phosphoinositides, and production of inosithol triphosphate (IP_3_) and diacyl glycerol (DAG), which in turn mobilize Ca^2+^ ions and activate phosphorylative processes that favor vesicle exocytosis. The involvement of CCR3 in the heteromeric assembly of CCRs favors the coupling to inhibitory G proteins reducing adenylyl cyclase (AC) activity and low cyclic adenosine monophosphate (cAMP) production. These events account for reduced vesicular exocytosis.

CCL5 is a 68-amino-acid protein that binds both pertussis toxin (PTx)-sensitive GPCRs (6, 8, 12) and Ptx-insensitive GPCRs (24) in the CNS. As to the central role of CCL5, the chemokine controls positively the mobilization of cytosolic Ca^2+^ and second messenger production in cultured neurons (25–28), astrocytes (29), and microglia (30), but it also activate GPCRs negatively coupled to adenylyl cyclase (AC)-mediated signaling, leading to the reduction of the endogenous level of cytosolic cyclic adenosine monophosphate (cAMP) (Figure 1) (25, 29).

## CCL5 Production in CNS

The endogenous level of CCL5 is very low, almost undetectable, in cerebral spinal fluid (CSF) of healthy individuals, but it increases dramatically when human immunodeficiency virus 1 (HIV-1) infection occurs (31–35), at the onset and during the progression of MS (9, 36–40). Increased central and peripheral CCL5 levels are also detected in mice suffering from the experimental autoimmune encephalomyelitis (EAE), an animal model reproducing most of the spinal cord pathological features of MS in humans [(41) and references therein]. Two are the mechanisms determining the dramatic increase of CCL5 bioavailability in CNS. First, the permeabilization of the blood–brain barrier that occurs in inflammation favors the entry of CCL5 from the periphery into the brain. Second, the concomitant massive local production of CCL5 from astrocytes and, to a lesser extent, from microglial cells triggered by pro-inflammatory citokines.

Microglia cells coordinate brain innate immunity, rapidly expand their population, and then migrate chemotactically to sustain inflammation and cells death. Microglia exist in the “resting” and the “activated” forms. In the resting state, that is an active surveying state, microglia cells in close proximity to neurons and astrocytes participate to the central network by releasing regulatory agents and by controlling homeostasis. In the “active” state, microglia releases pro-inflammatory effectors including TNF-alpha and IL-1beta, which diffuse to neighboring astrocytes, influencing their functions (42). These factors are the principal inducers of chemokine overproductions from astrocytes. Microglia filopodia make dynamic contact with astrocytes and neurons and have a pivotal role in controlling synaptic plasticity (43–45). The intimate contact of microglia with chemical synapses also makes the neuronal control of microglial functions possible. Actually, microglia cells are endowed with receptors for transmitters (46) the activation of which controls their activation state. Excitatory transmission favors the shift from the “resting” to the “activated” state of microglial cells, while inhibitory transmission reduces activation of microglial cells to the “resting” condition (47).

Astrocytes are innate immune sentinels that ensheath cerebral blood vessels controlling the entry of peripheral cells into the CNS parenchyma during infection. Astrocytoma cells as well as cultured astrocytes produce CCL5 upon incubation with the microglial effectors TNF-alpha or IL-1 beta, through an inflammatory-like response mediated by the p55 receptor-dependent signaling (42, 48). IFN-gamma from microglial cells, inactive on its own (42), seriously amplifies the effect of either TNF-alpha or IL-1 beta, worsening the pro-inflammatory signaling.

The expression and the release of CCL5 from astrocytes is tightly controlled by several receptor subtypes, including opioid receptors (49, 50), group III metabotropic glutamate receptors (2), alpha/beta noradrenergic receptors (51–53), and sphingosine-1-phosphate receptor (S1PR) subtype 1 (54), as well as by endogenous neurotoxin such as quinolinic acid acting at NMDA receptors (55). These receptors represent targets of therapeutics for inflammatory autoimmune disease typified by overexpression of CCL5.

Differently, data supporting the active production and release of the endogenous chemokine from neurons are lacking, suggesting that these cells do not release the chemokine.

## Expression of the Chemokine Receptors Targeted by CCL5 in Glial Cells

Once released in the synaptic cleft, CCL5 regulates the function of glial cells (microglia and astrocytes) themselves through autocrine processes.

The autocrine control of microglia functions is permitted by the existence of CCR5 receptors, and, to a less extent, of CCR1 and CCR3 receptors in these cells, which also permit the chemotactic movements of microglial cells during inflammation (56). CCR5s are also the receptors functional to HIV-1-induced pathogenetic mechanisms (57–59). As to the astrocytes, the existence of CCR1, CCR3, and CCR5 proteins in these cells has been a matter of discussion, and, despite some initial discrepancies (60), it was definitively demonstrated that adult astrocytes possess CCR1, CCR3, and CCR5 (61–63). CCR1, CCR3, and CCR5 proteins are expressed in cultured fetal astrocytes and in adult astrocytes from mammalian brains [(29), but see for a concise review Ref. (60)], as well as in astrocytic processes [gliosomes (64, 65)] isolated from the cortex and the spinal cord of adult mice (Table 1) (66). In particular, in physiological conditions, the expression of CCR5 in astrocytes is low but rapidly increases to abnormal pathological levels upon stimulation with TNF-alpha and by IL-1beta released by neighboring microglial cells, which causes persistent adaptation leading to a significant increase in the expression of most of the CCR proteins (55). This cascade of events occurs during inflammation, so that the overexpression of the receptors targeted by CCL5 is an event intimately linked to pathological conditions.

**Table 1:** Distribution of CCR1, CCR3, and CCR5 in astrocytes and neurons in the central nervous system (CNS) of mammals.

	Cell types	CNS region	Species	mRNA	Protein	Reference
CCR1	Neurons	Cultured neurons	Human	+		(67)
		Fetal brain	Macacque			(68)
		Cerebellum	Rat			(27)
		Hippocampus	Rat	+		(25)
		Neonatal and adult cerebellum	Rat	+		(69)
		Neonatal and adult cerebellum	Rat	+		(70)
		Cortical nerve endings	Human			(71)
		Cortical and spinal cord nerve endings	Mouse			(66, 72)
	
	Astrocytes	Neonatal brain	Mouse	+		(73)
		Neonatal brain	Mouse	+	+	(74)
		Fetal brain	Human		+	(75)
		Neonatal and adult cerebellum	Rat	+	+	(69)
		Neonatal and adult cerebellum	Rat	+	+	(70)
		Spinal cord gliosomes	Mouse		+	(66)

CCR3	Neurons	Cerebellar neurons	Rat		+	(27)
		Cortical neurons	Fetal human		+	(29)
			Fetal macaque		+	(29)
		Cortical nerve endings	Human		+	(71)
		Cortical and spinal cord nerve endings	Mouse		+	(66, 72)
		Fetal brain neurons	Human		+	(20)
	
	Astrocytes	Primary astrocytes	Human	+	+	(62)
		Fetal and adult astrocytes	Human, macaque	+	+	(76)
		Spinal cord gliosomes	Mouse		+	(66)
		Fetal brain astrocytes	Human		+	(20)

CCR5	Neurons	Neonatal DRG	Rat	+		(68)
		Cerebellar neurons	Rat		+	(27)
		Cortical neurons	Fetal human		+	(29)
			Fetal macaque		+	(29)
		Hippocampal neurons	Rat	+		(25)
		Embryonic neurons	Human	+		(26)
		Cortical nerve endings	Human		+	(71)
		Cortical and spinal cord nerve endings	Mouse		+	(66, 72)
		Neonatal brain	Rat	+	+	(48)
		Brain	Human		+	(33)
	
	Astrocytes	Cortical astrocytes	Fetal human		+	(29)
			Fetal macaque		+	(29)
		Fetal brain neurons	Human		+	(20)
		Fetal and adult astrocytes	Human		+	(76)
		Spinal cord gliosomes	Mouse		+	(66)
		Neonatal brain	Rat	+	+	(48)
		Brain	Human		+	(33)

## CCL5 and Glutamate Release from Astrocytes

In cultured astrocytes from both fetal simian and human brains, a significant increase in internal Ca^2+^ ions mobilization was observed upon exposure to CCL5 (29). Since intraterminal Ca^2+^ ions’ mobilization is a prerequisite to transmitter exocytosis also in these cells [(77) and references therein] the CCL5-mediated control of cytosolic Ca^2+^ bioavailability might suggest that the chemokine could favor/modulate glutamate exocytosis from astrocytes. Despite the expectation, however, the spontaneous and the depolarization-evoked release of [^3^H]d-aspartate ([^3^H]d-Asp) from gliosomal particles isolated from human brain tissue was not modified by CCL5 (29), compatible with the idea that activation of these receptors cannot modify the glutamate outflow of the excitatory aminoacid. Similarly, exposure of human and mouse glial particles [we refer to as gliosomes (64)] to CCL5 in the absence or in the presence of a concomitant depolarizing stimulus (i.e., 20 mM KCl-enriched solution, KCl substituting for an equimolar amount of NaCl) did not significantly modify the outflow of glutamate. This is compatible with the idea that the amount of Ca^2+^ ions mobilized in gliosomal cytosol by CCL5 acting at its own receptors was insufficient to prime vesicle docking and fusion with astrocyte membranes (66, 71). So far, data concerning the impact of CCL5 on glutamate uptake in microglia and astrocytes are not available and further investigations are needed to address these aspects.

## CCL5 in Neurons

### Expression of Chemokine Receptors Targeted by CCL5 in Neurons

Once released in the biophase, CCL5 also reaches neurons through a mechanism of volume diffusion, to modulate neuronal functions via paracrine mechanisms, mediated by CCRs express in neurons. This regulation eventually occurs when the external concentration of the chemokine is high and assures a sufficient diffusion of the agent in the synaptic space.

The first report of the existence of chemokine receptors in neurons dates to 1997 [(67), Table 1]. In this article, by combining immune-histochemical staining with receptor binding studies, the authors demonstrated that cultured human neuronal cells express CXCR2, CXCR4, CCR1, and CCR5 receptors. The authors suggested that these entities represent the binding site for the viral envelope protein gp120 of the HIV-1 virus on neuronal plasmamembranes, allowing a CD4-independent interaction between the virus and the neurons. Soon after, in 1998, Meucci et al. (25) demonstrated that cultured hippocampal neurons are endowed with several chemokine receptors, including CCR1 and CCR5 subtypes. In line with these observations, in1999, Klein et al. (29) provided clear evidence that a subpopulation of neurons in the cortex of human and macaque brains are endowed with CCR3, CCR5, and CXCR4 receptors. The existence of CCR1, CCR3, and CCR5 receptor proteins in neurons was then confirmed by other groups (20, 78–81), despite some discrepancies concerning their exact location (in the soma, on axonal processes and/or in nerve terminals). As to this aspect, a relevant finding was that CCR1, CCR3, and CCR5 receptor proteins exist in both human and rodent cortical nerve endings, as well as in rodent spinal cord terminals, i.e., in those parts of neuron where transmitter exocytosis occurs (66, 71, 72).

### CCL5 and Glutamate Release from Neurons

As observed in astrocytes, CCL5 controls the movement of Ca^2+^ ions in neurons (25, 29) but differently from astrocytes, this effect is sufficient to trigger changes in glutamate release efficiency. Based on these first observations, as well as on data published about a decade later (66, 71), the impact of CCL5 on glutamate release was found to represent a complex event, strictly dependent on the activity of the neurons themselves and on the region of the CNS under study.

When studying the changes in Ca^2+^ cytosolic bioavailability in hippocampal cultured neurons, Meucci et al. (25) showed that nanomolar CCL5 favored Ca^2+^ ion mobilization in resting condition but significantly reduced the increase in cytosolic Ca^2+^ that follows exposure of neurons to a depolarizing stimulus. Similarly, low nanomolar concentrations of the human recombinant CCL5 (hCCL5) exert opposite control on glutamate release [measured as the release of the unmetabolizable marker of glutamate, the compound [^3^H]d-Asp (82–85)] from nerve endings (synaptosomes) isolated from cortical specimens that were removed from consenting patients undergoing neurosurgery to reach deeply located tumors (71). In particular, hCCL5 elicited a significant increase in the spontaneous release of [^3^H]d-Asp from these terminals in basal conditions (i.e., in the absence of a depolarizing stimulus).

Facilitation of glutamate outflow relied on the activation of PTx-sensitive GPCRs positively coupled to phospholipase C (PLC)-mediated events, the activation of which led to the hydrolysis of membranes phosphoinositide and the mobilization of Ca^2+^ ions from Xestospongin-C-sensitive, inositol triphosphate (IP_3_)-dependent intraterminal stores located in the endoplasmic reticulum. Facilitation of glutamate release, however, turned to inhibition when hCCL5 was applied concomitantly to a mild depolarizing stimulus (i.e., 12 mM KCl). In this case, inhibition relied on the binding of hCCL5 to PTx-sensitive GPCRs negatively coupled to the AC/cAMP/protein kinase A (PKA) intraterminal enzymatic pathway (Figure 1). Comparable results were obtained when studying the impact of hCCL5 on human cortical slices. hCCL5 increased the basal release of [^3^H]d-Asp but significantly reduced the tritium overflow elicited by depolarizing stimuli (71).

Human recombinant CCL5-mediated facilitation of the spontaneous outflow of glutamate from both isolated nerve terminals and slices was prevented by MetRANTES, a broad-spectrum antagonist of the CCR1, CCR3, and CCR5 subtypes, confirming the involvement of these receptors (71). The impacts of the chemokine in the different experimental conditions (resting versus depolarized condition), however, seemed predictive of the existence of receptor subtype oligomers. Since antagonists able to discriminate among the different CCR subtypes were not available at that time, the pharmacological characterization of the receptor(s) accounting for the hCCL5-induced changes of glutamate outflow was carried out by pre-incubating human synaptosomes with antibodies raised against the N-terminal of the CCR1, the CCR3, and the CCR5 receptor proteins. By binding selectively to the outer side of the receptor protein, antibodies are expected to impede the interaction of the agonist with the orthosteric binding site, then mimicking receptor antagonists (86, 87). Pre-incubation of synaptosomes with antibodies raised against the extracellular NH_2_ terminals of CCR1 or of CCR5 receptor proteins impeded the hCCL5-induced facilitation of glutamate outflow from cortical nerve endings, while pre-treatment with anti-CCR3 was ineffective. Differently, hCCL5–mediated inhibition of glutamate exocytosis was prevented by pre-incubating synaptosomes with anti-CCR1, anti-CCR3 or anti-CCR5 antibodies, consistent with the view that different CCR oligomers account for the opposite effects observed.

The main criticism to the results obtained with human nerve terminals concerned the potential confounding factors originating from the origin of the human specimens, i.e., the brain of patients suffering from cerebral tumors. The receptor repertoire involved in the CCL5-mediated control of glutamate exocytosis in human specimens could have been altered because of the pathological overexpression of CCL5 in glioma cells. The effects of hCCL5 in human cortical synaptosomes, however, were soon after reproduced in glutamatergic nerve endings isolated from the cortex of mice, which represent healthy individuals, where the endogenous CCL5 level is expected to be low (88). Again, in mouse cortical terminals, the release of glutamate in basal condition (i.e., the absence of a depolarizing stimulus) was potentiated by CCL5, but the chemokine significantly inhibited the glutamate exocytosis evoked by a mild K^+^ depolarization (12 mM KCl) stimulus. The comparable effects observed in human and mice terminals allowed to conclude that the effects observed in human nerve endings were not influenced by the pathological origin of the tissue specimens.

Facilitation of the spontaneous release of glutamate as well as inhibition of 12 mM K^+^-evoked glutamate exocytosis from cortical synaptosomes was prevented by the selective CCR1 antagonist BX513 and by the selective CCR5 antagonist DAPTA, compatible with the involvement of CCR1/CCR5 heterodimers in the effect observed (66). Furthermore, the CCR3 antagonist, the compound SB 328437, failed to affect the CCL5-mediated facilitation of glutamate release in basal condition, but it strongly prevented the inhibitory effect exerted by the chemokine in depolarized nerve terminals. Comparable results could be drawn when using antibodies raised against the N-terminal of the CCR1, CCR3, and CCR5 receptor proteins, leading to conclude that (i) the receptor composition of the chemokine oligomers controlling glutamate release in mouse and human cortical nerve endings is largely conserved, (ii) the involvement of CCR3 in the oligomer expression dictates the coupling to inhibitory G proteins bridging negatively the chemokine receptor complex to the AC/cAMP/PKA transducing mechanism (Figure 1; Table 2), and (iii) the mouse brain tissue is appropriate to investigate the effects of CCL5 on central glutamatergic transmission (66).

**Table 2:** Correlation between the composition of CCR oligomers and the CCL5-mediated changes to glutamate release.

	Human cortical synaptosomes		Mouse cortical synaptosomes		Mouse spinal cord synaptosomes	
	Basal glutamate release	12 mM KCl-evoked glutamate overflow	Basal glutamate release	12 mM KCl-evoked glutamate overflow	Basal glutamate release	15 mM KCl-evoked glutamate overflow
CCR1	↑	↓	↑	↓	↑	↑
CCR3	Not involved	↓	Not involved	↓	Not involved	Not involved
CCR5	↑	↓	↑	↓	↑	↑

*The table resumes the modulatory action of CCL5 on the release of glutamate (measured as release of preloaded [^3^H]d-aspartate) correlating these events to the CCR subunits involved in the expression of chemokine oligomers targeted by the chemokine*.

*↓, inhibition of glutamet relesae; ↑, facilitation of glutamate exocytos*.

CCL5-mediated control of glutamate release in nerve terminals was not restricted to the cortex. The chemokine also efficiently modulates the release of glutamate from spinal cord nerve endings. However, differently from what observed in the cortex of adult mice, the spontaneous release of glutamate from spinal cord glutamatergic nerve endings was unaffected by nanomolar CCL5, while the depolarization-evoked glutamate exocytosis was significantly increased. Again, facilitation of glutamate exocytosis from these terminals involved CCR1/CCR5 heteromers positively coupled to PLC-induced IP_3_-mediated enzymatic pathway, leading to increased mobilization of Ca^2+^ ions in the cytosol. Also in this region, the receptor protein composition was clarified by using selective CCR antagonists as well as anti CCR antibodies recognizing the N-terminal of the receptor protein (66). These studies unveiled that CCR1/CCR5 heterodimers mediates the CCL5-induced facilitation of glutamate exocytosis, further confirming the hypothesis that CCR1/CCR5 oligomers preferentially couples to stimulatory G protein positively coupled to PLC-mediated events (Figure 1; Table 2). Notably, CCR3 immunoreactivity was detected in spinal cord synaptosomal lysates, but this receptor subtype was not involved in the CCL5-mediated effect.

## CCL5 in Demyelinating Disorders

The serum level of CCL5 was found to be significantly increased in patients suffering from MS (89–91) as well as in EAE animals (15, 41, 92). The highest levels of the chemokine were detected in the peripheral blood mononuclear cells (PBMCs) of MS patients suffering from the secondary progressive form of the disease, while lower level was observed in the PBMCs from patients with the relapsing–remitting form of MS (93). CCL5 levels are also increased in the CNS of MS patients as well as of EAE mice (9, 13, 22, 88, 93–100). The huge elevation of the CCL5 levels in CNS mainly depends on the increased peripheral production of the chemokine, but it also reflects the local overexpression of the chemokine in astrocytes activated by IL-1beta, TNF-alpha, and IFN-gamma (55), from neighboring microglia cells. All these observations are predictive of the role of CCL5 in the onset and progression of disease in MS patients. Accordingly, a CCL5 polymorphism [the CCL5-403 G/A single nucleotide polymorphism (22, 99)] is associated to a higher risk of susceptibility to the onset of the disease, while modified CCL5 ligands are efficacious in controlling the symptoms and the neurodegenerative processes in EAE mice (15).

CCR1, CCR3, and CCR5 exist in different cell types, including T lymphocytes, monocytes/macrophages, and immature dendritic cells, but also exist in neurons and astrocytes (101–104). In particular, CCR1 and CCR3 are expressed by circulating T cells as well as in monocytes, which are occasionally found in perivascular infiltrates in the brain of MS patients. Differently, CCR5-positive T cells and macrophages are concentrated in the active demyelinating lesions in CNS of MS patients (13, 22). To note, the expression of CCRs in CNS correlates with disease severity (105–107) as proved by the observation that clinical symptoms are reduced in CCR1, CCR3, or CCR5 knockout (k.o.) EAE animals, which also suggest redundancy in the chemokine system (7, 8, 99, 108).

## Glutamate in Demyelinating Disorders

Glutamate is the major excitatory neurotransmitter in CNS, where it mediates important physiological functions (i.e., synaptic plasticity, learning, and memory), but also triggers excitotoxic degenerative processes. Glutamate concentration in the synaptic cleft is finely tuned by several cellular mechanisms including active re-uptake and release from nerve terminals as well as presynaptic mechanisms of control mediated by auto- and/or heteroreceptors (65, 82, 109–116). Glutamate bioavailability is also affected by neighboring astrocytes [i.e., the cells that take up and release the aminoacid (77)] as well as by altered glutamate metabolism. l-Glutamate signaling, however, is not restricted to neuron/astrocyte compartments since glutamate receptors (GluR3-containing AMPA receptors and mGluR1/5 receptors) exist also in immune cells, including cells of the T lineage (117–119). Therefore, besides its role in controlling chemical transmission and excitotoxicity, glutamate may represent a chemo-attractant driving force for the recruitment and the migration of leukocytes and T cells into CNS site where glutamate release occurs (119).

Increased glutamate levels are found in the cerebrospinal fluid of MS patients (119–121) possibly because of the down-regulation of glutamate-metabolizing enzymes (glutamate dehydrogenase and glutamine synthase) and up-regulation of glutamate-producing enzyme glutaminase (122). In 2003, Sarchielli et al. (119) compared the levels of aspartate and glutamate in the CSF of patients suffering from different forms of MS and of controls healthy individuals. The authors find a significant increase of the glutamate levels in patients suffering from the relapsing–remitting form of MS. Interestingly, the glutamate levels were significantly higher in individuals suffering from the relapsing–remitting form of MS with active central lesions during the stable phase than in patients suffering from a similar form of disease, but without lesions. Inasmuch, high levels of glutamate were also detected in patients suffering from the secondary progressive form of MS.

Impaired glutamate bioavailability was also observed in EAE animals. However, depending on the animal model used and the brain region under study, opposite modifications of glutamate release efficiency were observed, consistent with the view that, in demyelinating disorders, impaired glutamate transmission at active synapses is a complex event. Increased glutamate release was detected in the spinal cord of EAE rats (123, 124) as well as in striatal and spinal cord nerve terminals of EAE mice (72, 88, 124–126), while reduced glutamate release was observed in cortical and hippocampal nerve endings of both mice and rats suffering from EAE disease (72, 88, 100, 126, 127). As to glutamate receptors, both metabotropic and ionotropic glutamate receptors (namely mGlu1/5 and mGlu4, mGlu2/3 receptors, and NMDA and AMPA receptors) control glutamate release (83, 109, 113, 115, 116, 128, 129). The expression and the function of these receptors were found to be altered in EAE mice when compared with controls (109, 128–136), suggesting that they represent suitable targets of drugs for the cure of MS symptoms Besides receptors, also glutamate transporter expression is modified in EAE rats (124), determining increased glutamate bioavailability (137) and consequent neurotoxicity.

Generalized ongoing subclinical axonal degeneration in lesioned and non-lesioned white matter, as well as in gray matter, is detectable in CNS of MS patients and seems to occur independently from inflammation or demyelination, representing an early cause of CNS damage in MS (138). Interestingly, besides the spinal cord, neurodegeneration also takes place in other brain regions, such as the cortex and the hippocampus, and could be responsible of cognitive and affective dysfunctions (121, 139–141) that represent common and early manifestations of MS (142, 143).

## CCL5-Mediated Control of Glutamate Release in EAE Mice

The thesis that central chemokines and classic transmitters functionally cross-talk in CNS has several implications and add new aspects of interest to the role of chemokines in the synaptic derangements that typify neuro-inflammatory central disease.

As already stated, an abnormal overproduction of CCL5 in the spinal cord, and to a lesser extent in the cortex, of EAE mice was evidentiated in immunocytochemistry analysis and confirmed in tissue homogenate (88, 100, 126) and in blood (88). Concomitantly, changes in glutamate exocytosis from nerve endings isolated from the cortex and the spinal cord of EAE mice were observed. Quite interestingly, the modifications of glutamate exocytosis observed in cortical and spinal cord synaptosomes from EAE mice recall the presynaptic modulation elicited by CCL5 in nerve terminals in healthy mice. Actually, glutamate exocytosis was reduced in cortical nerve endings from EAE mice [i.e., in this CNS region, CCL5 inhibits glutamate exocytosis in control animals (66, 71)], but it was drastically increased in spinal cord terminals [where a positive role of CCL5 on glutamate release was observed in healthy animals (66)].

In both CNS regions, the altered glutamate exocytosis was paralleled by impaired second messenger production. Again, the alterations in cAMP ad IP_3_ productions observed in both cortical and spinal cord synaptosomal subpopulations from EAE mice were reminiscent of the modulatory presynaptic effects exerted by CCL5 on the corresponding enzymatic pathways in nerve terminals from control, non-immunized, mice. In fact, endogenous cAMP was drastically reduced in cortical synaptosomes but not in the spinal cord, where IP_3_ production, the second messenger accounting for the CCL5-mediated presynaptic facilitation of glutamate exocytosis was significantly augmented (Figure 1) (66, 88).

The changes in second messenger productions and release efficiency could be explained by assuming that the prolonged *in vivo* CCR activation elicited by the high CCL5 could have triggered adaptive intraterminal changes in nerve terminals (144–146), which are retained in “*ex vivo, in vitro*” synaptosomal preparations and can emerge in “*in vitro*” functional studies as changes in glutamate release efficiency and second messenger production. As a matter of fact, the abnormal expression of the chemokine could have reverberated on the CCR repertoire presynaptically located on glutamatergic nerve terminals, leading to adaptation of the CCRs heteromers controlling glutamate exocytosis. These adaptations might lead to changes in the receptor expression and/or associated signaling that might account for the profound changes in glutamate exocytosis in nerve terminals from EAE mice. The CCR composition of the presynaptic chemokinergic oligomers involved in the CCL5-mediated control of glutamate exocytosis in both cortical and spinal cord nerve endings of EAE mice, however, was conserved when compared to control mice, indirectly suggesting that adaptation in CCR subunits assembly were not involved in the EAE-induced changes to the CCL5-mediated control of glutamate exocytosis described above (72).

As to the second messengers, the strict correlation linking CCL5 levels, glutamate release efficiency and IP3, and cAMP accumulation was confirmed by the observation that administration of drugs able to reduce the overexpression of CCL5 in CNS [i.e., the antidepressant desipramine (DMI)] (126) restored both presynaptic functions (i.e., transmitter exocytosis as well as second messenger production) at glutamatergic nerve endings in the cortex of mice suffering from EAE. The beneficial effects exerted by DMI were mediated by the change in noradrenaline bioavailability, due to blockade of the noradrenaline transporters. Actually, the transient increase in the endogenous amine in the synaptic cleft elicited by DMI activates the α and β receptors expressed in astrocytes in the near proximity of the noradrenergic nerve terminals, the activation of which hampers the central endogenous production and release of pro-inflammatory chemokines, including CCL5 (53, 126, 147) from these cells. Interestingly, the peripheral production of the chemokine (which at that stage of disease is already augmented) was unaffected (126).

To conclude, hampering the central overproduction of CCL5 leads to a marked amelioration of the presynaptic defects in terms of release of glutamate and second messenger production. Altogether, these observations clearly support a strict correlation between the increased production of CCL5 in the CNS and the onset of synaptic glutamatergic alteration in EAE mice, also strengthening the pathological role of the “glial to neuron” “CCL5–glutamate” interaction.

## CCL5 in MS: Clinical Studies

In 2016, Centonze et al. (40) demonstrated that the endogenous concentration of CCL5 in the cerebrospinal fluid of MS patients suffering from the active form of the disease was largely increased when compared to healthy individuals and to patients at the inactive stage of disease. Inasmuch, the authors showed a significant correlation between the endogenous level of CCL5 and the amount of IL-1 beta, used as a marker of gravity of the disease. CSF levels of RANTES were associated with enhanced cortical excitability in the cortex, as suggested by results from studies in which cortical excitability and plasticity was monitored with transcranial magnetic stimulation in MS patients. In these experiments, the authors highlighted a high correlation between the increased intracortical facilitation and the endogenous amount of CCL5. Differently, no correlation emerged when studying the relation linking CCL5 and a long-term potentiation-like synaptic plasticity measured through theta burst stimulation in the same patients. Despite the contrasting observation, the authors concluded that CCL5 couples inflammation and synaptic excitability in MS brains.

## Impact of Disease-Modifying Drugs on the Endogenous Production of CCL5 in Demyelinating Disorders

As already stated in this review, CCL5 production is increased in MS patients suffering from both the remitting and the non-remitting form of the disease (15, 41, 89–92). Interestingly, most of the therapeutics currently in use for the cure of MS reduces significantly the overexpression of CCL5.

It is the case of interferon-beta-1b (IFN-beta-1b); administration of this drug prevents CCL5 overproduction in the sera, in the peripheral blood and in the adherent mononuclear cell supernatants during both the relapse and the remission stage of the pathology. These observations are compatible with the idea that CCL5 might be involved in determining the molecular events accounting for the action of IFN-beta-1b in MS patients (38).

Similarly, glatiramer acetate, an approved drug for the treatment of MS, was reported to reduce the TNF-alpha-induced CCL5 mRNA overexpression in human U-251 MG astrocytes. This effect was attributed to the inhibition of mRNA transcription and led to the conclusion that glatiramer acetate may exert its therapeutic effect in MS also by inhibiting pro-inflammatoy signaling (148).

Activation of cannabinoid receptors, which represents a therapeutic approach useful to control the progression of central neuroinflammation in EAE mice and MS patients, also reduces the endogenous availability of the chemokine CCL5 being concomitantly beneficial to the progression of the demyelinating disorder (41, 149).

Laquinimod is a novel orally administered drug for the treatment of relapsing–remitting MS. The molecular events accounting for its therapeutic effects are far from to be elucidated. Monocytes obtained from laquinimod-treated patients tended to secrete lower levels of the pro-inflammatory chemokines CCL2 or CCL5 (150).

Another orally active disease-modifying drug is Fingolimod. Fingolimod is a pro-drug, rapidly metabolized to its active form, the fingolimod-phosphate (fingolimod-P). By acting at the S1PRs in microglia cells, in circulating T cells, and in the spleen, fingolimod-P prevents the egress of lymphocytes and exerts central anti-inflammatory effects favoring remyelination (151). Recent data demonstrated that *in vivo* oral (the drug dissolved in the drinking water) administration of this drug largely ameliorated the clinical symptoms in EAE mice. The treatment was beneficial to the inflammation and demyelination in the spinal cord of EAE mice, also significantly reducing the endogenous content of CCL5 in this CNS region (100).

Quite interestingly, the abovementioned therapeutics were also described to ameliorate glutamatergic synaptic transmission (152–155) further supporting the strict connection linking CCL5 overexpression and glutamatergic synaptic derangements.

## Conclusion

**Figure 2:**
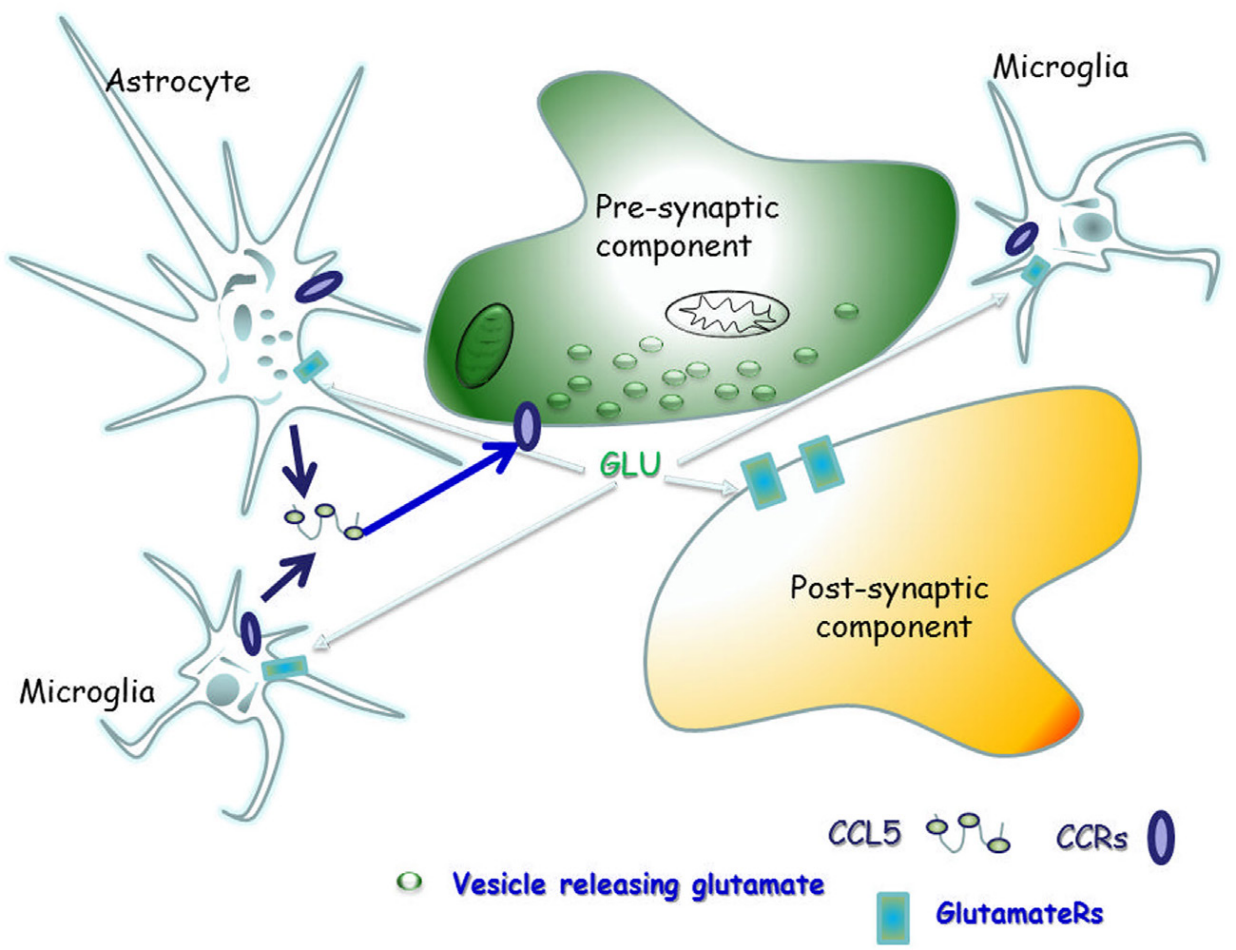
The “quad partite” synapse is a functional structure consisting of neurons, astrocytes, and microglia cells. It represents the simplest central Unit, where adaptive and damaging processes occur in neuro-inflammatory disorders, including the demyelinating one. The concept originates from the tripartite synapse (152), but it is characterized by a highest level of complexity, since microglia is included as players of synaptic derangement. In a simplified model for demyelinating disorder, microglia cells migrating from periphery to central nervous system (CNS) as well as resident central microglia rapidly expand their populations and differentiate into the M1- and M2-cell subgroups, which exert various and mostly opposite functions in the brain. Microglia cells of the M1 group releases pro-inflammatory cytokines, including CCL5, which in turn activate astrocytes. In a whole these events sustain and worsen central inflammatory processes. Differently, M2 microglia secretes anti-inflammatory cytokine and its neuroprotective. Astrocytes are the most abundant glial cells in the human brain and represent the innate immune sentinels that sheath cerebral blood vessels controlling the entry of peripheral cells into the CNS during infection. Astrocytes are neuroprotective at the initial stage of disease, since they reduce local hyper-glutamatergicity by active glutamate uptake processes. Astrocyte activation, however, becomes pathological upon prolonged exposure to the pro-inflammatory compounds released from neighboring microglial cells. At this stage, reactive astrocytes become hypertrophic, do not uptake efficiently glutamate, but release much more cytokines (including CCL5), which accelerate neurodegenerative processes. CCL5 actively released by activated astrocytes and microglia by one side and the abnormal bioavailability of glutamate in the synaptic cleft, on the other side, reverberate onto neurons, eliciting structural and functional changes at chemical synapses.

The scope of this manuscript is to review the literature concerning the physio-pathological role of CCL5 in controlling glutamate transmission in the CNS of healthy mammals, as well as of individuals and animals suffering from demyelinating disease, in order to highlight the main role of CCL5 as a modulator of the neuroimmune cross-talk in the “quad partite” synapse in CNS (Figure 2).

These effects, together with the well-known chemo-attractant role of the chemokine toward glial cells, suggest that CCL5 exerts a dual role in the CNS of individuals suffering from MS. On one hand, the chemokine impairs the chemical transmission at asymmetric synapses in selected region of the CNS. On the other hand, it worsens the course of disease progression by favoring the recruitment of pro-inflammatory glial cells in the site of the lesion.

When considering its role as modulator of glutamate transmission, the chemokine preferentially emerges as a key effector of the “astrocytes to neurons” signaling in CNS. Actually, the chemokine released from astrocyte and microglia is an efficient paracrine modulator of glutamate release at synaptic boutons of glutamatergic neurons in both healthy and demyelinating conditions, while its autocrine role of modulator of glutamate overflow from astrocytes is less evidnet (Table 1).

Therapeutic approaches aimed at containing the overexpression of the chemokine might represent therefore a useful approach to the cure of MS.

## Author Contributions

The author confirms being the sole contributor of this work and approved it for publication.

## Conflict of Interest Statement

The author declares that the research was conducted in the absence of any commercial or financial relationships that could be construed as a potential conflict of interest.
